# Impact of high-risk fertility behaviours on underfive mortality in Asia and Africa: evidence from Demographic and Health Surveys

**DOI:** 10.1186/s12884-021-03780-y

**Published:** 2021-05-01

**Authors:** Rafi Amir-ud-Din, Lubna Naz, Aneela Rubi, Muhammad Usman, Umesh Ghimire

**Affiliations:** 1 Department of Economics, COMSATS University Islamabad, Lahore Campus, Lahore, Pakistan; 2 Department of Economics, Karachi University, Karachi, Pakistan; 3 Research Scholar, Department of Economics, COMSATS University Islamabad, Lahore, Pakistan; 4 Department of Management Sciences, COMSATS University, Islamabad, Lahore Campus, Lahore, Pakistan; 5 New ERA, Kalopul, Rudramati Marga, Kathmandu, 44600 Bagmati Nepal

**Keywords:** Underfive mortality, High-risk fertility behaviours, women’s age at childbirth, Birth spacing and birth order, Demographic and health survey

## Abstract

**Background:**

Maternal age < 18 or > 34 years, short inter-pregnancy birth interval, and higher birth order are considered to be high-risk fertility behaviours (HRFB). Underfive mortality being disproportionately concentrated in Asia and Africa, this study analyses the association between HRFB and underfive mortality in selected Asian and African countries.

**Methods:**

This study used Integrated Public Microdata Series-Demographic and Health Surveys (IPUMS-DHS) data from 32 countries in sub-Saharan Africa, Middle East, North Africa and South Asia from 1986 to 2017 (*N* = 1,467,728). Previous evidence hints at four markers of HRFB: women’s age at birth of index child < 18 or > 34 years, preceding birth interval < 24 months and child’s birth order > 3. Using logistic regression, we analysed change in the odds of underfive mortality as a result of i) exposure to HRFB individually, ii) exposure to any single HRFB risk factor, iii) exposure to multiple HRFB risk factors, and iv) exposure to specific combinations of HRFB risk factors.

**Results:**

Mother’s age at birth of index child < 18 years and preceding birth interval (PBI) < 24 months were significant risk factors of underfive mortality, while a child’s birth order > 3 was a protective factor. Presence of any single HRFB was associated with 7% higher risk of underfive mortality (OR 1.07; 95% CI 1.04–1.09). Presence of multiple HRFBs was associated with 39% higher risk of underfive mortality (OR 1.39; 95% CI 1.36–1.43). Some specific combinations of HRFB such as maternal age < 18 years and preceding birth interval < 24 month significantly increased the odds of underfive mortality (OR 2.07; 95% CI 1.88–2.28).

**Conclusion:**

Maternal age < 18 years and short preceding birth interval significantly increase the risk of underfive mortality. This highlights the need for an effective legislation to curb child marriages and increased public investment in reproductive healthcare with a focus on higher contraceptive use for optimal birth spacing.

**Supplementary Information:**

The online version contains supplementary material available at 10.1186/s12884-021-03780-y.

## Background

Child mortality is a serious global health issue. Although underfive mortality decreased by 59% from 93 deaths per 1000 live births in 1990 to 39 in 2018, 5.3 million children died before their fifth birthday in 2018 [1]. Sub-Saharan Africa (sSA) has the highest mortality rate in the world and contributes 52% of all underfive deaths, followed by Central and Southern Asia, accounting for 29% of underfive mortality [2]. Sustainable Development Goal 3 seeks to reduce neonatal mortality to 12 per 1000 live births and underfive mortality to 25 per 1000 live births [3].

Several socioeconomic factors, including mother’s age and hereditary characteristics, nutritional status and substance use, were associated with increased child mortality [4]. Lack of skilled human resources, inadequate infrastructure and low investment in health systems have significantly increased the rate of child and maternal mortality in low and middle-income countries (LMICs) [5, 6]. Among various risk factors of underfive mortality, women’s age at the time of birth, interpregnancy interval and child’s birth order have particularly been highlighted as some of the most critical risk factors behind underfive mortality [7–9].

Recently, there has been a growing realisation that a woman may simultaneously experience multiple risk factors of underfive mortality, also called high-risk fertility behaviours (HRFB). HRFB can be expressed in terms of women’s age at birth being too early or too late, shorter birth intervals and higher numbers of live births [10, 11]. Evidence suggests that HRFB is widespread and a significant cause of neonatal and underfive mortality in LMICs [12, 13]. Bangladesh Demographic and Health Survey (DHS) showed that 41.8% of women showed HRFB, out of whom 33% showing any single HRFB and 8.8% multiple HRFB [11]. A study from Ethiopia indicated a higher underfive mortality was associated with maternal age 15 to 18 years and having repeated pregnancies with shorter interpregnancy spacing [14].

LMICs face severe socioeconomic and demographic challenges. They also face issues inextricably linked with underfive mortality such as child marriage, high fertility and high population growth. Around 46% of women in South Asia and 37% in sSA were married before their 18^th^ birthday [15]. Teenage pregnancy rate in Bangladesh is as high as 35% [16]. According to 2018 estimates, global fertility rate in Bangladesh was 2.5 and 4.7 in sSA [17]. Global annual population growth rate in 2019 was 1% as compared with 2.7% in sSA [18].

Given multiple risk factors of underfive mortality interacting with each other in a complicated way in LMICs, this study sought to analyze links between HRFB and underfive mortality using a large sample of 32 countries in sSA, Middle East, North Africa and South Asia in the period of over three and half decades. Analysis of specific combinations of various HRFBs may highlight structural drivers and inform evidence-based policy for targeted actions against underfive mortality.

## Methods

### Data sources

We used data from the Integrated Public Use Microdata Series project of Demographic and Health Surveys (IPUMS-DHS) for this study [19]. DHS collects information on critical demographic and health-related indicators such as mortality rates, fertility and family planning using a stratified sample of households based on cluster design with an average response rate of more than 90%. Advantages of using IPUMS-DHS data are that all variables are consistently coded across all countries and survey periods. IPUMS-DHS databases include data about the individual respondents and household information is linked from household recodes. IPUMS-DHS database has data for 35 countries from sSA, Middle East, North Africa and South Asia from1986 to 2017.

We restricted our sample to only those countries with available information about underfive mortality and HRFB in at least one survey wave. According to this criterion, 32 countries from sSA, Middle East, North Africa and South Asia from1986 to 2017 were selected. Sample size was 1,467,728 from 142 survey waves with as little as one survey from Angola and ten from Senegal.

### Study outcomes

Outcome variable is underfive mortality which refers to death of all children under the age of five. Underfive mortality in this study is coded as binary variable: index children who died before reaching the fifth birthday were coded as 1 and those who were alive at the time of the mother’s interview and had not yet reached the age of five were coded as 0.

### Exposures

Exposure variables are HRFBs of the mother, measured by four parameters: mother’s age < 18 at birth of the index child; mother’s age > 34 at birth of the index child; birth interval < 24 months after the preceding birth up to index birth; and birth order of index child > 3.

In addition to the four indicators of HRFB individually, we constructed dichotomous variables i.e., *any single high-risk fertility behaviour*, which took the value 1 if any single risk factor (mother’s age at birth < 18 or > 34 years or PBI < 24 months or birth order > 3) was present, and 0 otherwise. Our next variable of interest was a multicategory variable *multiple high-risk fertility behaviour* where the absence of any high-risk fertility behaviour (coded as 0) was compared with the presence of any single high-risk fertility behaviour (coded as 1) and multiple high-risk fertility behaviours (coded as 2).

Following the literature [10], we also tested seven specific combinations of HRFB: i) mother’s age at birth < 18 years and birth interval < 24 months; ii) mother’s age at birth < 18 years and birth order > 3; iii) mother’s age at birth < 18 years and birth interval < 24 months and birth order > 3; iv) mother’s age at birth > 34 years and birth interval < 24 months; v) mother’s age at birth > 34 years and birth order > 3; vi) mother’s age at birth > 34 years and birth interval < 24 months and birth order > 3; and vii) birth interval < 24 months and birth order > 3.

### Potential confounders

Though many studies have identified a wide array of risk factors of underfive mortality such as size of child at birth [20] and women empowerment [21], antenatal care attendance [22], our criterion for including confounding variables was that they should be associated with both HRFB and underfive mortality. As only those confounding factors can be appropriate for this study which occurred *before* HRFB and by implication before birth of the index child, size of child at birth, antenatal care attendance and women’s empowerment are, therefore, not included in the list of confounding factors.

Recently, a vast literature has emerged, linking breastfeeding with underfive mortality [23]. However, in the context of DHS data, a child could be reported as “never breastfed”, because it died young (especially in case of neonatal death). With regard to women’s empowerment, this, strictly speaking, is not a binary outcome because it keeps on changing and is a function of several factors which include the birth of her children [24]. Domestic violence against infertile women [25] is just one example how children affect women’s life circumstances. As the birth of a child may influence women’s life circumstances (for example, birth of male children increases mother’s bargaining power within the household especially in some parts of South Asia), mother’s empowerment may suffer from endogeneity, and has thus been excluded from the list of potential confounders.

Given the preceding debate on confounding, we have adjusted our model with father’s and mother’s education, father’s and mother’s occupation, urban/rural residential status, household wealth status and country and time fixed effects. Parental education variables were constructed with no education as reference and primary, secondary and higher education as the alternative categories. Parental occupation variables were dichotomous, with “not currently working” as reference and “currently working” as alternative. Residential status is also a dichotomous variable with rural residence as reference and urban residence as alternative. Household wealth status is a quintiles index variable with the poorest quintile as reference and poorer, middle, richer and richest quintiles as the alternative categories. We adjusted the models with time fixed effects by categorising the survey years from 1986 to 2017 into three periods: 1986–2000, 2001–2010 and 2011–2017.

### Statistical analysis

We used the logistic regression model to estimate the association between HRFB and underfive mortality. We analysed how exposure to HRFB affects the odds of underfive mortality in four different ways: i) exposure to HRFB individually, ii) exposure to any single HRFB risk factor; iii) exposure to multiple HRFB risk factors; and iv) exposure to specific combinations of HRFB risk factors.

Additionally, we did sub-group country-level analysis by estimating the impact of i) mother’s age at birth of the index child < 18 or > 34 years, ii) mother’s age at birth of the index child < 18 *and* > 34 years separately, iii) preceding birth interval (PBI) < 24 months and iv) birth order of index child > 3 on underfive mortality (Figs. 2, 3, 4 and 5). We regressed these variables on underfive mortality using logistic regression for each country separately and estimated adjusted odds ratios of these variables. Each model was adjusted for father’s and mother’s education, father’s and mother’s occupation, urban/rural residential status, household wealth status and country and time fixed effects. We used Stata’s command idpover to make forest plots for each country in the sample. Odds ratios of all countries were averaged to give the overall effect of different measures of HFRB on underfive mortality.

Since DHS has a complex survey design, sampling weights are required to correct for the bias in probability selection [26]. Regression analysis was, therefore, done after adjusting for sampling design (stratification and clustering) using “svy” command in Stata/MP 15.1.

### Eligibility criteria

Women aged 15–49 years who gave birth in the 5 years preceding the interview were included in this analysis. Only singleton births were included because twins have different mortality risks for both children and mother [27, 28]. The sample includes all singletons born to a woman because restricting the sample to only last born of the singleton birth children would give mortality rates that are significantly different from WHO underfive mortality rates [29].

## Results

**Fig. 1:**
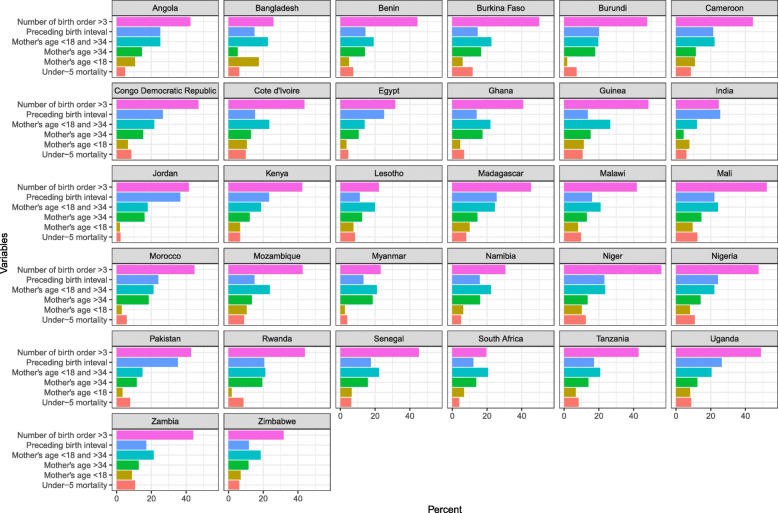
Rates of underfive mortality and various indicators of high-risk fertility behaviours. Source: IPUMS-DHS

Underfive mortality rates revealed significant geographical and temporal differences over the number of surveys in each country (Fig. 1). Average underfive mortality in 32 countries from 1986 to 2017 was 77 per 1000 live births. Underfive mortality rates in our sample were 93 per 1000 live births during 1986–2000, 77 during 2001–2010 and 52 during 2011–2017. Jordan had the lowest underfive mortality rate with 22 per 1000 live births and Niger the highest with 125 per 1000 live births. Approximately 7% of women gave birth before they reached their 18th birthday with the highest percentage concentrated in Bangladesh (17.5%) and the smallest in Burundi (1.7%). Approximately 14% of women gave birth when they were above 34, with the highest percentage in Rwanda (19.4%) and the smallest in India (4.4%). Around 21% of the women < 18 years or > 34 years gave birth, with the highest rate in Guinea (26.4%) and the lowest in India (12%). Around 20% of children had a preceding birth interval < 24 months, with the highest in Jordan (36.6%) and the lowest in Lesotho (11.2%). Over 40% of the children had birth order > 3. The lowest percentage of such children was in India (25%) and the highest in South Africa (56%).

### Individual high-risk fertility behaviours

Compared to the reference category of mothers whose age at birth of the index child was ≥18 years, mothers who were < 18 years had higher odds of underfive mortality (aOR 1.61; 95% CI 1.56–1.67), unlike women > 34 years at birth of the index child as compared to mothers aged ≤34 (aOR 0.98; 95% CI 0.96–1.01; Table 1). Compared to the reference category of women with PBI ≥24 months, those with PBI < 24 months had higher odds of underfive mortality (aOR 2.02; 95% CI 1.97–2.07; Table 1). Compared with children with birth order ≤3, children with birth order > 3 had lower odds of underfive mortality (aOR 0.91; 95% CI 0.89–0.93; Table 1).

**Table 1:** High-risk fertility behaviours: individual risk factors

	(1)	(2)	(3)	(4)
	aOR^a^	aOR	aOR	aOR
**Mother’s age at birth**				
≥18 years	1			
< 18 years	1.61^***^			
	(1.56,1.67)			
**Mother’s age at birth**				
≤34 years		1		
> 34 years		0.98		
		(0.96,1.01)		
**Length of PBI**				
≥ 24 months			1	
< 24 months			2.02^***^	
			(1.97,2.07)	
**Child’s birth order number**				
First, second, or third born				1
> 3				0.91^***^
				(0.89,0.93)
**Mother’s education**				
No Education	1	1	1	1
Primary	0.92^***^	0.92^***^	0.91^***^	0.91^***^
	(0.89,0.94)	(0.90,0.95)	(0.88,0.94)	(0.89,0.94)
Secondary	0.74^***^	0.74^***^	0.73^***^	0.73^***^
	(0.71,0.77)	(0.71,0.77)	(0.69,0.76)	(0.70,0.76)
Higher	0.56^***^	0.55^***^	0.55^***^	0.53^***^
	(0.51,0.62)	(0.50,0.61)	(0.48,0.63)	(0.48,0.58)
**Father’s education**				
No Education	1	1	1	1
Primary	0.94^***^	0.93^***^	0.90^***^	0.92^***^
	(0.91,0.96)	(0.91,0.96)	(0.87,0.93)	(0.89,0.95)
Secondary	0.86^***^	0.86^***^	0.85^***^	0.85^***^
	(0.83,0.89)	(0.83,0.89)	(0.82,0.89)	(0.82,0.88)
Higher	0.75^***^	0.74^***^	0.78^***^	0.74^***^
	(0.70,0.80)	(0.70,0.79)	(0.72,0.84)	(0.69,0.79)
**Mother’s working status**				
Currently not working	1	1	1	1
Working	1.09^***^	1.07^***^	1.13^***^	1.09^***^
	(1.06,1.12)	(1.05,1.10)	(1.09,1.16)	(1.06,1.11)
**Father’s working status**				
Currently not working	1	1	1	1
Working	0.98	0.97	1.00	0.97
	(0.89,1.07)	(0.88,1.06)	(0.89,1.11)	(0.88,1.06)
**Residential status**				
Rural	1	1	1	1
Urban	0.96^**^	0.95^**^	0.96^*^	0.94^***^
	(0.93,0.99)	(0.92,0.98)	(0.92,0.99)	(0.91,0.97)
**Household wealth status**				
Poorest	1	1	1	1
Poorer	0.99	0.99	0.99	0.99
	(0.97,1.02)	(0.97,1.02)	(0.96,1.02)	(0.97,1.02)
Middle	0.92^***^	0.92^***^	0.94^***^	0.94^***^
	(0.90,0.95)	(0.89,0.95)	(0.90,0.97)	(0.91,0.97)
Richer	0.90^***^	0.90^***^	0.94^**^	0.91^***^
	(0.87,0.93)	(0.87,0.93)	(0.91,0.98)	(0.88,0.94)
Richest	0.76^***^	0.75^***^	0.77^***^	0.77^***^
	(0.72,0.79)	(0.71,0.78)	(0.73,0.82)	(0.73,0.80)
**Country fixed effects**	Yes	Yes	Yes	Yes
**Time fixed effects**	Yes	Yes	Yes	Yes
N	901,934	901,934	748,896	1,000,229
F	172.0	157.9	168.3	155.0
p	0	0	0	0

Exponentiated coefficients; 95% confidence intervals in brackets

^*^ *P <* 0.05, ^**^ *P <* 0.01, ^***^ *P <* 0.001

^a^Adjusted odds ratio

### Any single high-risk fertility behaviours

With regards to the reference category of women without HRFB, any single HRFB (mother’s age < 18 or > 34 or PBI < 24 months or birth order > 3) was associated with higher odds of underfive mortality (aOR 1.07; 95% CI 1.04–1.09; Table 2). Increased education of both father and mother, as well as increased household wealth status were significantly associated with lower odds of underfive mortality. In contrast, maternal occupation was associated with higher odds (aOR 1.07; 95% CI 1.04–1.10), but father’s occupation status as well as residential status of the child had no significant impact on the odds of underfive mortality.

**Table 2:** High-risk fertility behaviours: no HFRB versus any single risk factor

	(1)	
	adjusted OR	95% CI
**Any single high-risk category**		
No	1	
Any single risk factor	1.07^***^	(1.04,1.09)
**Mother’s education**		
No Education	1	
Primary	0.93^***^	(0.90,0.96)
Secondary	0.77^***^	(0.74,0.81)
Higher	0.57^***^	(0.51,0.63)
**Father’s education**		
No Education	1	
Primary	0.93^***^	(0.90,0.96)
Secondary	0.86^***^	(0.83,0.89)
Higher	0.75^***^	(0.70,0.81)
**Mother’s working status**		
Currently not working	1	
Working	1.07^***^	(1.04,1.10)
**Father’s working status**		
Currently not working	1	
Working	0.93	(0.84,1.02)
**Residential status**		
Rural	1	
Urban	0.97	(0.93,1.00)
**Household wealth status**		
Poorest	1	
Poorer	0.99	(0.97,1.03)
Middle	0.96^*^	(0.93,1.00)
Richer	0.92^***^	(0.88,0.95)
Richest	0.77^***^	(0.74,0.81)
Country fixed effects	Yes	
Time-fixed effects	Yes	
N	819,471	
F	130.7	
*p*	0	

Exponentiated coefficients; 95% confidence intervals in brackets

^*^ *P <* 0.05, ^**^ *P <* 0.01, ^***^ *P <* 0.001

### Multiple high-risk fertility behaviours

Compared with women who did not show any risky fertility behaviour, both women with single HRFB (aOR 1.07; 95% CI 1.05–1.09) as those with multiple HRFB were associated with higher odds of underfive mortality (aOR 1.39; 95% CI 1.36–1.43). See further details in Additional File 1.

### Specific combinations of risks of high-risk fertility behaviours

Mother’s age < 18 years at birth of the index child and PBI < 24 months were both associated with higher odds of underfive mortality (aOR 2.07; 95% CI 1.88–2.28; Table 3). The same applied to mother’s age < 18 years at birth of the index child, PBI < 24 months and birth order > 3 (aOR 1.997; 95% CI 1.10–3.62).

**Table 3:** High-risk fertility behaviours: specific combinations of risk factors

	(1)
	adjusted OR
Age at birth < 18 years and birth interval < 24 months	
No	1
Yes	2.07^***^
	(1.88,2.28)
Age at birth < 18 years and birth order > 3	
No	1
Yes	1.82^*^
	(1.11,3.01)
Age at birth < 18 years and birth interval < 24 months and birth order > 3	
No	1
Yes	2.00^*^
	(1.10,3.62)
Age at birth > 34 years and birth interval < 24 months	
No	1
Yes	1.93^***^
	(1.83,2.05)
Age at birth > 34 years and birth order > 3	
No	1
Yes	0.98
	(0.95,1.01)
Age at birth > 34 years and birth interval < 24 months and birth order > 3	
No	1
Yes	1.95^***^
	(1.84,2.06)
Birth interval < 24 months and birth order > 3	
No	1
Yes	1.82^***^
	(1.76,1.88)
Country fixed effects	Yes
Time-fixed effects	Yes

Exponentiated coefficients; 95% confidence intervals in brackets

^*^P < 0.05, ^**^ *P <* 0.01, ^***^ *P <* 0.001

Mother’s age > 34 years at birth of the index child and PBI < 24 months were also associated with higher risks of underfive mortality (aOR 1.93; 95% CI 1.89–2.05). PBI < 24 months and birth order > 3 were also associated with higher risks of underfive mortality (aOR 1.82; 95% CI 1.77–1.88). Though mother’s age > 34 years at birth and birth order > 3 had no significant impact, mother’s age > 34 years at birth of index child, birth order > 3 and PBI < 24 months were associated with higher risks of underfive mortality (aOR 1.95; 95% CI 1.84–2.07). This suggests that PBI < 24 months was the most important risk factor of underfive mortality.

**Fig. 2:**
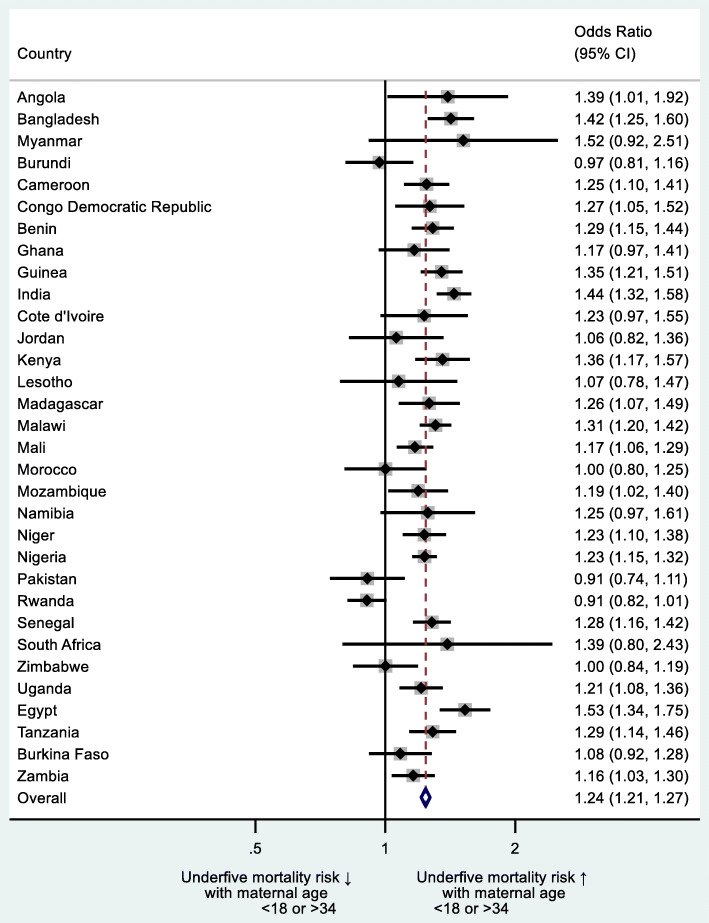
Country-level odds ratios: mother’s age at birth of index child < 18 or > 34 years as risk factor of underfive mortality

Out of 32 countries, a statistically significant positive association existed between mother’s age < 18 or > 34 years at birth of the index child and underfive mortality in 19 countries only (Fig. 2). Overall, odds of underfive mortality were higher for mothers whose age was < 18 or > 34 years as compared to mothers aged 18 to 34 years (aOR 1.24; 95% CI 1.21–1.27). The highest risk of underfive mortality was observed in Egypt (aOR 1.53; 95% CI 1.34–1.75) and smallest in Zambia (aOR 1.16; 95% CI 1.03–1.30).

**Fig. 3:**
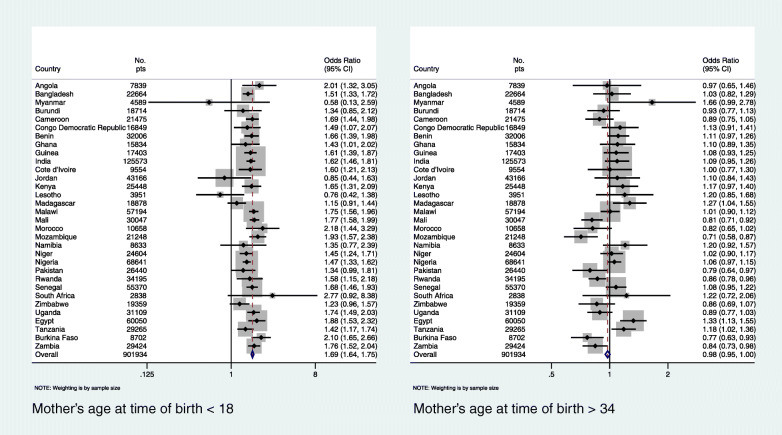
Country-level odds ratios: mother’s age at birth of index child < 18 or > 34 years as risk factor of underfive mortality

Figure 3 gives country-level ORs of underfive mortality regressed separately on mother’s age < 18 *and* > 34 years at birth of index child. In 23 out of 32 countries, maternal age < 18 years was associated with significantly higher odds of underfive mortality compared to women aged ≥18. Maternal age > 34 years, however, was a significant risk factor of underfive mortality in only three countries, while it statistically significant protected against underfive mortality in six countries.

**Fig. 4:**
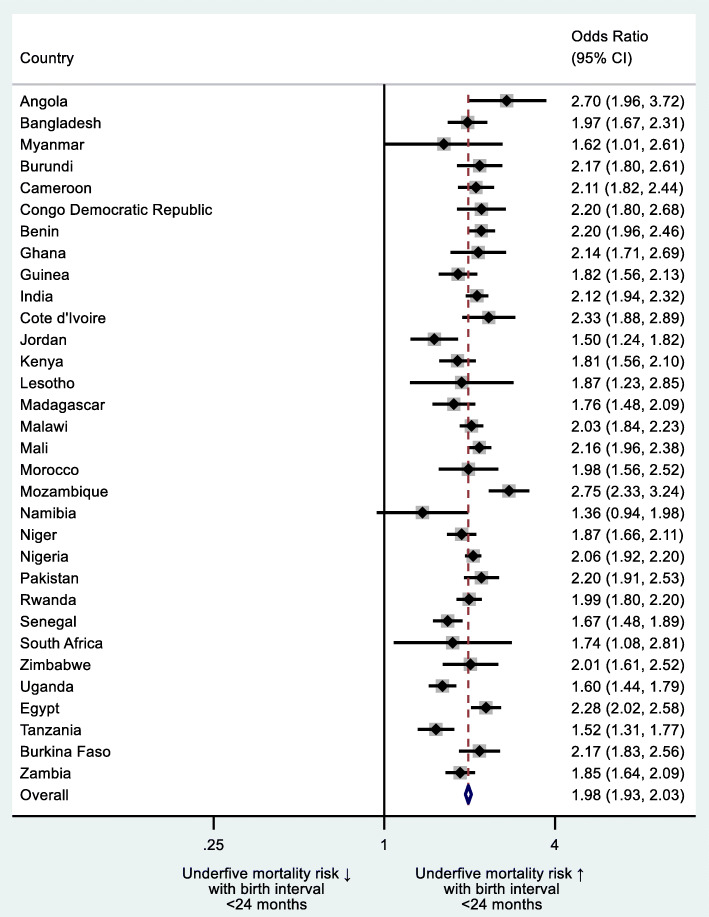
Country-level odds ratios: preceding birth interval (PBI) < 24 months as risk factor of underfive mortality

In 31 of the 32 countries, PBI < 24 months significantly increased the risk of underfive mortality (Fig. 4). Overall, odds of underfive mortality were higher for children with PBI < 24 months as compared to PBI ≥24 (aOR 1.98; 95% CI 1.93–2.03). The highest risk with PBI < 24 months was observed in Mozambique (aOR 2.75; 95% CI 2.33–3.24) and the lowest in Jordan (aOR 1.50; 95% CI 1.24–1.82).

**Fig. 5:**
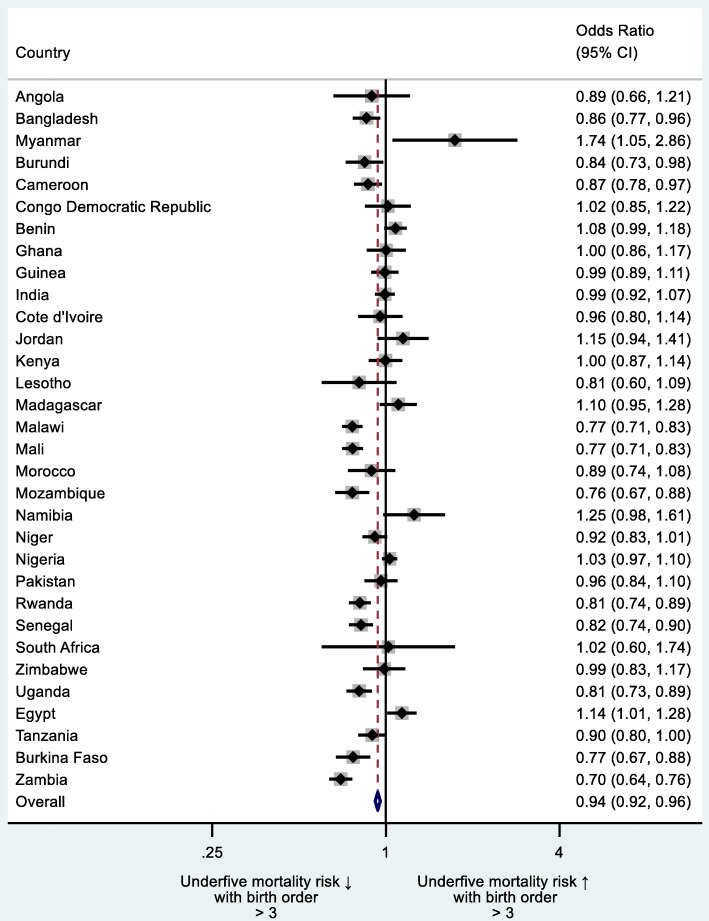
Country-level odds ratios: child’s birth order > 3 as risk factor of underfive mortality

Birth order > 3 significantly decreased the risk of underfive mortality in 11 countries with only increased risks in two (Fig. 5). No significant association between birth order > 3 and underfive mortality was found in 19 countries. Overall, odds of underfive mortality were lower for children whose birth order was > 3 (aOR 0.94; 95% CI 0.92–0.96) with the lowest risk in Zambia (aOR 0.70; 95% CI 0.64–0.76) and the highest in Myanmar (aOR 1.74; 95% CI 1.05–2.86).

## Discussion

Underfive mortality rates revealed significant geographical and temporal differences. Average underfive mortality from 32 countries was 77 per 1.000 live births from 1986 to 2017, from a lowest rate in Jordan (22 per 1.000 live births) to a highest in Niger (125 per 1.000 live births). Average underfive mortality rates decreased from approximately 93 per 1000 live births during 1986–2000 to 77 during 2001–2010 and 52 during 2011–2017.

Mother’s age < 18 years at birth of the index child and PBI < 24 months were significant risk factors of underfive mortality, while birth order > 3 was protective.

Our major finding of mother’s age < 18 years being a risk factor of underfive mortality is consistent with many previous studies linking young mothers with high underfive mortality [30–34]. Underfives born to young mothers are vulnerable to malnutrition and morbidity, leading to higher risks of mortality than those born to adult mothers. Young mothers are not fully matured physically and deprived of nutritional and biological advantages, which directly affect foetal development [35]. Young mothers have higher risks of adverse birth outcomes, such as low birth weight, stunting and infant mortality [36–38]. Young mothers are less experienced in child care than older ones and their children may be more vulnerable to unintentional injuries. Such injuries, the third leading cause of underfive mortality in the world, include drowning, traffic accidents, accidental asphyxia, poisoning and falls [39]. In the US maternal age between 15 to 24 years was associated with a significantly higher risk of sudden unexpected infant death than maternal age > 30 years [40]. In a study in Pakistan children born to mothers who were married as minors were significantly more likely to suffer from repeated episodes of diarrhoea than those born to women married as adults and diarrhoea has been found to be one of the leading causes of underfive mortality [41].

Women married at younger ages have little decision-making power with respect to their health and that of their children in LMIC [42]. Child brides have smaller bargaining power within households compared with their adult counterparts [43]. As women are not allowed to visit a doctor without a male family member in some contexts [44], access to healthcare may be difficult when women married young [44]. In some resource-constrained settings, girl brides may themselves have limited access to adequate nutrition, which may, in turn, result in reduced foetal nutrition and breastfeeding [45, 46]. Consequently, combined effects of foetal malnutrition and suboptimal breastfeeding may increase underfive mortality.

PBI < 24 months appeared to be the strongest predictor of underfive mortality in our study, in accordance with many previous studies [7, 31, 47–50]. Different mechanisms have been proposed to explain this. The maternal depletion hypothesis suggests that with short PBIs women cannot recover their nutritional stores which may result in malnutrition in the next pregnancy [51]. Malnutrition may result in depletion of folate stores and folate insufficiency is associated with increased risks of anaemia, stunted intrauterine growth, preterm birth and neural tube defects [52–54]. Faced with malnutrition, women’s bodies are known to prioritise their own needs over the nutritional needs of the foetus [55].

The sibling rivalry hypothesis is another theoretical explanation that short PBIs are associated with the risk of underfive mortality. This sibling hypothesis suggests that closely spaced children compete for attention of the parents as well as for scarce resources in the household, resulting in a weakened immune system, increased risks of infectious diseases and mortality among children [56]. Low birthweight, stunted growth of the neonate as well as decreased quality and quantity of breastmilk may mediate between PBI and underfive mortality [57].

Short PBIs are associated with increased risks of premature rupture of membranes, antepartum haemorrhage, anaemia, placental abruption, which ultimately may increase the risk of stillbirth and neonatal mortality [53, 58]. Depression of the mother may also increase the risk of subsequent mortality of infants [59].

Evidence suggests that older siblings have a crucial role as caretakers of younger ones [60]. A study in Australia found that supervision lapses, which were partly due to indoor and outdoor household duties and talking/socialising. Lack of supervision was the major reason behind fatal unintentional drownings of children [61].

Birth order > 3 was protective against underfive mortality in our study. This finding contradicts the dominant view that higher birth order increases the risk of underfive mortality [48, 62–65]. Evidence also suggests an inverse-U shaped relationship between birth order and underfive mortality, with second-born and third-borns having the highest risks [9].

Though lower age of the mother in high parity births could be a risk factor of underfive mortality, it is plausible to think that mothers learn from the experience of bringing up older children helping them take care of higher birth order children more effectively [66]. Parenting difficulties of first-born children such as sleep deprivation are also well documented [67]. Experienced parents may cope more effectively with parental stress than first-timers [68].

Women in LMIC are often married young and the majority of them stay-at-home. Most of them are relatively young even after giving birth to a fourth and fifth child. This hypothesis is corroborated by our data: mean age of women giving birth to five children in our sample is 32 years. Also maternal age > 34 years was not associated with significantly increased risks of underfive mortality. The relatively young maternal age, even when having high parity, may explain the protective effect of higher-order births against underfive mortality.

Higher mortality among children of higher birth order may also be explained by less access to healthcare than first or second-born children [69]. Health services in most LMIC, however, have been expanding over the time, making access to healthcare for children of higher birth order easier, leading to lower odds of underfive mortality for high parity births [70].

Some studies, in contrast with our findings, showed older maternal age as risk factor of underfive mortality [71, 72]. In our study very few women gave birth at age > 34 years (14%) and even less than 3% after 40 [45]. Some specific combinations of HRFB such as maternal age < 18 years and PBI < 24 months significantly increased the odds of underfive mortality.

This study has some limitations. Different geographic regimes have different social, cultural and economic dynamics. Various health vulnerabilities and country- and region-specific characteristics of the participants could be a potential source of bias. As exposure variables were based on interviews and self-reported accounts occurring in the previous 5 years, a likelihood of recall bias may have been present. Further, causality between risk factors and underfive mortality cannot be assessed because the data utilized in our study is of cross-sectional in nature.

## Conclusions

This study analysed associations between HRFB and underfive mortality using DHS data from 32 countries. Younger maternal age and short PBI significantly increased the risks of underfive mortality. In contrast, higher birth order was associated with lower risk. Maternal age > 34 years did not turn out to be a risk factor. HRFB analysis also showed that presence of any single factor of HRFB as well as combinations of factors of HRFB significantly increased the risk of underfive mortality. These findings highlight the need for effective legislation to curb teenage marriages and increased public investment in reproductive healthcare with a focus on contraceptive use for an optimal birth spacing.

## Supplementary Information

**Additional file 1.** High-risk fertility behaviours: no HRFB versus single versus multiple risk factors

## Data Availability

The datasets generated and analysed during the current study are available subject to permission from the DHS program, in the (IPUMS-DHS) repository (https://www.idhsdata.org/idhs/index.shtml).

## Abbreviations

aOR: adjusted Odds Ratio
CI: Confidence Interval
HRFBs: High-risk fertility behaviours
DHS: Demographic and Health Survey
LMICs: Low- and middle-income countries
PBI: Preceding birth interval
SDGs: Sustainable Development Goals
UNIGME: United Nations Inter-Agency Group for Child Mortality Estimation
USAID: United States Agency for International Development
