# Supplementary material for: Impact of high-risk fertility behaviours on underfive mortality in Asia and Africa: evidence from Demographic and Health Surveys
Source: BMC Pregnancy Childbirth. 2021 May 1;21:344. doi: 10.1186/s12884-021-03780-y (PMC8088561; doi:10.1186/s12884-021-03780-y)
Supplement: Supplementary file 1 — Additional file 1. High-risk fertility behaviours: no HRFB versus single versus multiple risk factors [file 12884_2021_3780_MOESM1_ESM.docx]

**Additional File**

Additional File 1: High-risk fertility behavior: no HRFB versus single versus multiple risk factors

|  | (1) |  |
| --- | --- | --- |
|  | adjusted OR | 95% CI |
| **High-risk fertility behavior** |  |  |
| No | 1 |  |
| Single high-risk category | 1.07^***^ | (1.04,1.09) |
| Multiple high-risk category | 1.39^***^ | (1.35,1.43) |
| **Mother’s education** |  |  |
| No Education | 1 |  |
| Primary | 0.94^***^ | (0.92,0.97) |
| Secondary | 0.78^***^ | (0.75,0.81) |
| Higher | 0.58^***^ | (0.53,0.64) |
| **Father’s education** |  |  |
| No Education | 1 |  |
| Primary | 0.93^***^ | (0.91,0.96) |
| Secondary | 0.87^***^ | (0.85,0.91) |
| Higher | 0.76^***^ | (0.71,0.81) |
| **Mother’s working status** |  |  |
| Currently not working | 1 |  |
| Working | 1.06^***^ | (1.04,1.09) |
| **Father’s working status** |  |  |
| Not currently working | 1 |  |
| Working | 0.98 | (0.90,1.07) |
| **Residential status** |  |  |
| Rural | 1 |  |
| Urban | 0.95^***^ | (0.92,0.98) |
| **Household wealth status** |  |  |
| Poorest | 1 |  |
| Poorer | 0.99 | (0.97,1.02) |
| Middle | 0.94^***^ | (0.92,0.97) |
| Richer | 0.91^***^ | (0.88,0.94) |
| Richest | 0.77^***^ | (0.74,0.80) |
| Country fixed effects | Yes |  |
| Time-fixed effects | Yes |  |
| N | 1000229 |  |
| F | 171.6 |  |
| p | 0 |  |

Exponentiated coefficients; 95% confidence intervals in brackets

^*^ *P <* 0.05, ^**^ *P <* 0.01, ^***^ *P <* 0.001
